# Research themes and key data points for child and adolescent emergency department mental health presentations: A national Delphi study

**DOI:** 10.1111/acem.15056

**Published:** 2024-12-02

**Authors:** Marietta R. John‐White, Edmund Proper, Frank Muscara, Franz E. Babl, Vicki A. Anderson, Catherine L. Wilson, Meredith L. Borland, Bruce J. Tonge, Kylie M. Gray, Glenn A. Melvin, Amit Kochar, Rohan Borschmann, Richard Haslam, Emma J. Tavender, Michael S. Gordon, Stuart R. Dalziel, Karen Smith, Simon S. Craig, the Paediatric Research in Emergency Departments International Collaborative (PREDICT) Network

**Affiliations:** ^1^ Department of Emergency Medicine Monash Medical Centre Melbourne Victoria Australia; ^2^ Department of Paediatrics, School of Clinical Sciences Monash University Melbourne Victoria Australia; ^3^ Clinical Sciences Murdoch Children's Research Institute Melbourne Victoria Australia; ^4^ Department of Emergency Medicine Royal Children's Hospital Melbourne Victoria Australia; ^5^ Department of Paediatrics The University of Melbourne Melbourne Victoria Australia; ^6^ Melbourne School of Psychological Sciences The University of Melbourne Melbourne Victoria Australia; ^7^ Murdoch Children's Research Institute Melbourne Victoria Australia; ^8^ Department of Emergency Medicine Perth Children's Hospital Perth Western Australia Australia; ^9^ School of Medicine The University of Western Australia Perth Western Australia Australia; ^10^ Department of Psychiatry Monash University Melbourne Victoria Australia; ^11^ University of Warwick Coventry UK; ^12^ Centre for Social and Early Emotional Development (SEED), School of Psychology Deakin University Melbourne Victoria Australia; ^13^ Monash University Melbourne Victoria Australia; ^14^ Department of Acute Care Medicine University of Adelaide Adelaide South Australia Australia; ^15^ Department of Emergency Medicine Women's and Children's Hospital Adelaide South Australia Australia; ^16^ The University of Melbourne Melbourne Victoria Australia; ^17^ Department of Psychiatry University of Oxford Oxford UK; ^18^ Curtin University Perth Western Australia Australia; ^19^ Health Services Research Unit Royal Children's Hospital Melbourne Victoria Australia; ^20^ Early in Life Mental Health Service Monash Health Melbourne Victoria Australia; ^21^ Department of Surgery and Paediatrics The University of Auckland Auckland New Zealand; ^22^ Emergency Department Starship Children's Health Auckland New Zealand; ^23^ Department of Epidemiology and Preventive Health Monash University Melbourne Victoria Australia; ^24^ Department of Research and Innovation Silverchain Melbourne Victoria Australia; ^25^ Ambulance Victoria Melbourne Victoria Australia

**Keywords:** adolescent, emergency, mental health, research prioritization

## Abstract

**Objective:**

The objective was to identify a prioritized list of research themes and key data points (baseline data and research outcomes) for future studies regarding child and adolescent emergency department (ED) mental health presentations.

**Methods:**

A prospective survey‐based Delphi process was undertaken in Australia within the Pediatric Research in Emergency Departments International Collaborative (PREDICT) network. Hospital‐based and community‐based clinicians, researchers, police, ambulance paramedics, pediatric patients, and their carers were recruited to generate research themes and key data points for future pediatric ED mental health research. Responses were collated and analyzed by a steering group consisting of pediatric mental health, medical, and research/academic experts. Participants then prioritized the items through three survey rounds using a 9‐point Likert‐type scale to generate a final prioritized list.

**Results:**

184 participants (36 patients/carers and 148 clinicians/researchers) were recruited and generated 267 items for initial prioritization; 23 completed all survey rounds. The surveys identified a consensus of 71 items: 35 research themes and 36 key data points (11 baseline data points and 25 research outcomes) for future research. The top‐rated research themes included patient/staff safety within the ED, the efficacy of dedicated mental health spaces, and the importance of patient follow‐up. Important baseline data points included risk factors for mental health presentations and history of child abuse and/or family violence. Top‐rated research outcomes included the occurrence of severe behavioral disturbance in the ED, the use of parenteral sedation, and ED re‐presentation and/or suicide attempt postdischarge.

**Conclusions:**

The Delphi process identified a prioritized list of research themes and key data points that will inform future research on child and adolescent mental health‐related ED presentations.

## INTRODUCTION

Over the past 10–15 years, there has been a progressive increase in child and adolescent emergency department (ED) mental health presentations, including for self‐harm, depression, and behavioral disorders^1^, ^2^ in Australia. Between 2010 and 2014, there was a mean increase of 27% in adolescents presenting with self‐harm and/or suicidal ideation to New South Wales EDs.^3^ In the state of Victoria, there was a year‐on‐year increase of 3.5% in pediatric mental health presentations, with an even greater increase amongst those aged 10–14 years, between 2008–2009 and 2014–2015.^4^

The COVID‐19 pandemic has exacerbated this progressive increase, with significant growth in ED presentations for eating disorders, self‐harm, and anxiety‐related disorders in the pediatric population^5^ since 2020. This has been a global phenomenon and persisted after the first years of the pandemic, with similar findings reported in other countries.^6^, ^7^

Research prioritization is critical to effectively assign limited acute care resources. Multiple pediatric emergency medicine (PEM) research networks worldwide have published consensus research priorities to guide future multicentre projects.^8^, ^9^, ^10^, ^11^, ^12^, ^13^, ^14^ However, the growing challenge of mental health presentations has rarely been addressed.^8^, ^9^, ^10^, ^11^, ^12^, ^13^, ^14^ In view of the recent increase in child and adolescent mental health presentations, we believed it necessary to determine research priorities for this population.

With the input of young people and their parents/carers, hospital‐ and community‐based clinicians, police officers, and researchers, our aims were to:

1. Identify the highest priority research themes relating to emergency care for children and adolescents with mental health presentations and
2. Generate a list of key data points (addressing both baseline data and important research outcomes data) that should be collected to guide future research.

## METHODS

### Study design

We conducted a four‐stage Delphi survey in Australia to gather and prioritize items for Aims 1 and 2. The Delphi process is a well‐validated method involving repeated surveys.^15^ This removes the inherent group‐conformity bias prevalent in face‐to‐face meetings by maintaining anonymity of respondents.^15^ Participants (clinicians, researchers, patients, and families as outlined below) were initially invited to provide free‐text research themes/questions and key data points via a semistructured survey. These responses were then collated and refined by an expert steering group with input from an advisory body.

The expert steering group consisted of pediatric emergency physicians, pediatricians, child psychologists and psychiatrists, a biostatistician, a knowledge translation researcher, and a research coordinator. The advisory body included consumers with lived experience of pediatric ED mental health presentations, members of police and ambulance, a psychologist, and an Indigenous Australian representative. The advisory body provided input into study design, participant recruitment, language used in the surveys, and data interpretation.

The surveys were designed and administered using the secure, web‐based software platform REDCap,^16^, ^17^ hosted and managed by Monash University Helix. The study was approved by the Monash Health Research Ethics Committee (HREC/58487/MonH‐2020‐200419) prior to the commencement of the surveys.

### Participants

Requests for expressions of interest for emergency health care providers to participate in the project were distributed to Australian hospitals and emergency service organizations using links and contacts of the Pediatric Research in Emergency Departments International Collaborative (PREDICT) network.^18^ A variety of settings were targeted (12 participating hospitals across three Australian states: Victoria, Queensland, and South Australia), spanning rural/regional centers, tertiary pediatric hospitals, and nontertiary metropolitan hospitals. Recipients were requested to distribute study information to care providers, and to recruit patients and parents/carers from their local ED.

Survey respondents were recruited in two streams:

1. Care providers and researchers: emergency clinicians (doctors, nurses, psychologists) and community‐based emergency response personnel (ambulance paramedics, police officers), and
2. Care recipients: patients and their parents/carers who have a lived experience of receiving ED pediatric mental health care.

#### Recruitment of care providers

*Care providers* were included in the study if they had provided care for a patient under 18 years of age who had presented to an ED with mental health concerns in the previous 6 months. A mental health–related presentation was defined as any ED visit with a triage presenting complaint or an ED discharge diagnosis relating to mental health as well as any visit that required mental health clinician referral, defined according to the International Statistical Classification of Diseases and Related Health Problems, Tenth Revision, Australian Modification (ICD‐10 AM) codes relating to mental and behavioral disorders (F01‐F99), emotional state, appearance and behavior (R45.0‐R46.8), and/or a diagnosis consistent with intentional self‐harm (X71‐X83).

Research leads at each site disseminated a hard copy or email invitation to hospital‐based care providers. The invitation included a link/QR code for care providers to register for the study. Community services such as police officers and ambulance paramedics were also recruited by advertising within the respective organizations once statewide organizational approval was granted for their members to participate in the study. The advertisement included a QR code that they could scan to register in the study.

#### Recruitment of care recipients

Participants who had *received* care (patients aged <18 years, parents/carers) were considered eligible if they or their child had a mental health–related ED presentation during the 2‐month recruitment period. We did not set a minimum age for participation and did not exclude youth with disabilities. We aimed to recruit a minimum of 20 patients/carers, 20 clinicians, and 20 other community‐based care providers. However, we did not have a set ratio of health care providers to patients/carers and aimed to recruit as many as possible from the participating health services.

All surveys were conducted in English and included two reminders sent at weekly intervals. Participants who required interpreter services in the ED were excluded. Children under 16 years of age were required to provide parent/carer consent; as such, those under 16 years of age who did not wish for their parent/carer to be informed of their ED presentation were also excluded.

Eligible participants were identified by their treating clinician and provided with a brief flyer about the study as well as a “permission to contact” form and/or a QR code to register their interest. They were invited to participate once the form was completed.

We did not seek formal written consent. Consent was implied after individual participants registered their interest, provided contact details, and completed at least the first survey.

### Data collection

#### Stage 1A: Item generation

The first survey requested general demographic data from each participant and provided targeted, semistructured questions to encourage generation of future research questions and key data points. It was developed in two sections: the first section invited respondents to identify *research questions* for pediatric ED mental health presentations in general as well as within specific subgroups of ED presentations (suicide attempt and self‐harm, acute behavioral disturbance, drug and alcohol misuse, eating disorders, neurodevelopmental disorders, anxiety and emotional disturbance) and whether there were additional specific considerations for Aboriginal, Torres Strait Islander, and Māori children. A second section invited participants to highlight *key data points* (baseline data and/or outcomes) they believed were important to measure. The recruitment of participants and completion of survey one occurred concurrently over 2 months, from May to July 2022.

#### Stage 1B: Collation and refinement of initial results

Once free‐text results were available from the first survey, research themes were collated, and duplicate entries were removed by two steering group members (MRJW and SSC) independently. Any medical terminology was simplified into lay language at this point. We did not perform a literature review to determine whether or not research questions had already been answered and did not restrict any topics as being “out of scope.” Key data points were divided into outcome measures and baseline characteristics and grouped into specific topic areas.

#### Survey Round 2: Prioritization

Once results had been collated, Survey Round 2 (circulated in September–October 2022) presented participants with a comprehensive list of research themes/questions and key data points, grouped by topic (e.g., eating disorders, suicidality). We did not provide additional explanatory text for any research theme/key data point. Participants were asked to rate the importance of each item (research themes/question or key data point) on a 9‐point Likert‐type scale. Each end of the scale was labeled (1 = not important and 9 = extremely important); there were no labels for other points. There was also opportunity for participants to provide free‐text comments under each choice to explain their reasoning, although this was rarely utilized. Participants were offered a $50AUD voucher on completion of the second round survey.

At the conclusion of the survey, there was a free‐text box that allowed participants to add any topics (research themes/questions and/or key data points) that had not been mentioned in the survey. The responses were again collated by the steering group members with input from the advisory group and included in the next round of the survey.

Items ranked in Survey Round 2 were eligible for inclusion in the following surveys if they met both the following criteria, defined a priori before data were collected and based on previous guidelines for the generation of core outcome measures^19^:

1. Rated 7–9 by >50% of participants from both care provider and those receiving care and
2. Rated 1–3 by no more than 15% of participants from both groups.

If responses did not meet the above criteria, they were excluded from future surveys.

#### Survey Round 3: Reprioritization

Items satisfying the inclusion criteria at the end of Survey Round 2 were included in Survey Round 3 and sent to respondents for repeat prioritization (November–December 2022), using the same 9‐point Likert‐type scale. Each item was presented to respondents alongside the mean score from both participant groups in Survey Round 2. This survey round also included new free‐text suggestions from Survey Round 2 if they were different to the questions and/or outcomes presented in earlier survey rounds.

Survey Round 3 aimed to develop a finalized list of research questions/themes and key data points, with items selected if they met consensus criteria based on the results from Survey Round 3. We defined, through investigator consensus and based on previous recommendations, consensus *agreement* as: (1) 70% or more of participants scored the Item 7–9 and 15% or less of participants scored the Item 1–3 in both the care provider and care receiver groups or (2) 90% or more participants scored the Item 7–9 from either stakeholder group.^19^ We defined consensus *disagreement* as 70% or more of participants scored the Item 1–3 and 15% or less of participants scored the Item 7–9 in both the care provider and care receiver groups or (2) 90% or more participants scored the Item 1–3 from either stakeholder group.^19^ Those who did not meet either of these criteria were deemed *inconclusive results*.

#### Stage 4: Analysis of inconclusive results

After two stages of prioritization, a fourth survey was required to rescore nine items (six research questions/themes and three key data points) that had received inconclusive results from Stage 3.

Data are presented using percentage of respondents. Outcomes are separated into those that occur during an ED visit and those that occur after discharge from the ED.

## RESULTS

**FIGURE 1:**
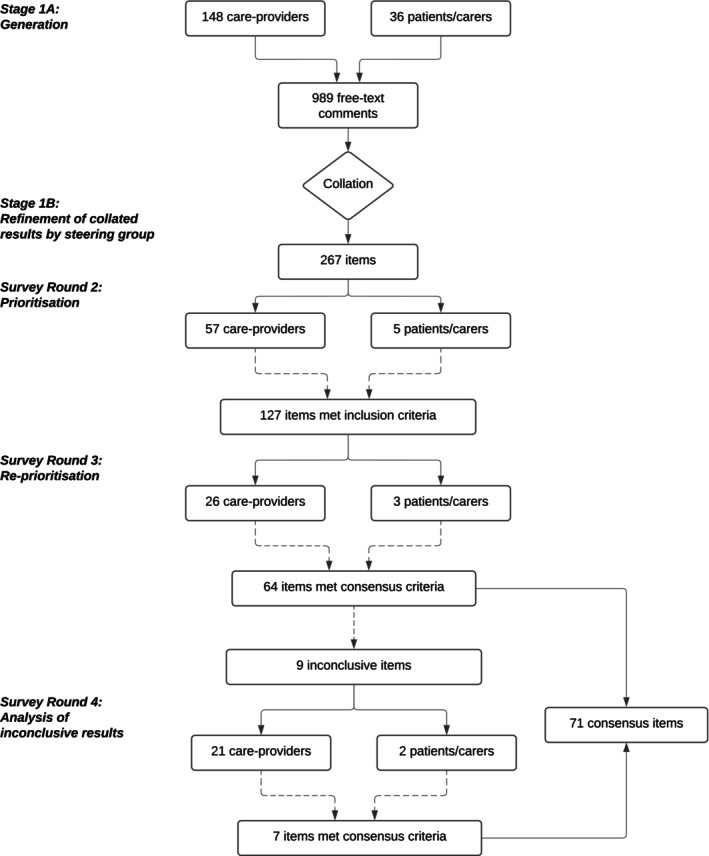
Delphi process for generation and prioritization of items, including Stage 4 for analysis of inconclusive items from previous surveys.

A total of 184 participants—36 care recipients (patient/carer, seven identified as young people, 19 as parent/carers, and 10 did not provide information) and 148 care providers (Table 1)—responded to the first round of the survey and produced 989 free‐text comments. The most common reasons for patients/carers to have presented to the ED at the time of study recruitment were suicidal ideation/self‐harm as reported by 10 participants and/or “mental health concerns” as reported by eight participants. Three participants presented for eating disorders and two for overdose, and two other presentations were for vomiting and acute onset of obsessions and compulsions. Of the 23 patient/carer respondents who provided information on their ED presentations for mental health concerns in the preceding 12 months, 11 had presented to ED with mental health concerns once or twice, eight had presented three to five times, three had presented six to 10 times, and two more than 10 times. These 989 individual free‐text responses were then collated into 267 items (187 research questions/themes, 21 baseline variables, and 59 outcomes). Of the 267 items in survey round two, 127 items met criteria for reprioritization in Survey Round 3 (see Figure 1), and six new questions were added from free‐text responses.

**TABLE 1:** Demographic characteristics of study participants.

	Patient/carer^a^	Care providers^a^
Participants for each survey round		
Round 1	36 (100)	148 (100)
Round 2	5 (14)	57 (39)
Round 3	3 (8)	26 (18)
Round 4	2 (6)	21 (14)
Self‐reported ethnicity (for Round 1 participants)		
Aboriginal/Torres Strait Islander	3 (8)	2 (1)
Australian	18 (50)	101 (68)
English/Irish/Welsh/Scottish	3 (8)	27 (18)
Chinese	0	5 (3)
Other	4^b^ (11)	10^c^ (7)
Ethnicity not reported	8 (22)	3 (2)
Professional group (Round 1 participants)		
Nursing staff		55
Paramedics		35
Emergency clinicians		32
Psychologist or other mental health clinician		10
Police/protective services officers		9
Psychiatrist		3
Security staff		1
Other		3^d^
Median age (years)		
Young person (*n* = 7)^e^	16	
Parent/carer (*n* = 19)^e^	44	
Number of ED presentations with mental health concerns in the past 12 months^f^		
1–2	11	
3–5	8	
6–10	3	
>10	2	

*Note*: Data are reported as *n* (%).

^a^ Percentage refers to the percentage of Round 1 respondents.

^b^ Other ethnicity (patient/carer group): Greek, Samoan, Venezuelan, Māori.

^c^ Other ethnicity (care provider group): New Zealand/Indian, Russian, African, Indian, Malaysian, Persian, Sri Lankan, Greek, Italian.

^d^ Other professional group: family violence advisor, social worker, medical administration.

^e^ 10 patient/carers did not indicate whether they were a young person or a parent/carer.

^f^ No response provided by 12 patient/carer respondents.

Survey Round 3 identified 64 items priority items, with an additional nine items considered inconclusive and required rescoring in survey four; seven of these items were included in the finalized consensus list giving a total of 71 items (Figure 1). These included 35 *research questions/themes* and 36 *key data points* (11 baseline data items and 25 outcomes).

The top‐rated research questions/themes related to safety (for both staff and patients), the efficacy of dedicated mental health treatment spaces, and the effectiveness of adequate follow‐up to prevent re‐presentation (Table 2). The top four listed questions/themes were: (1) What is required for an ED to be considered a safe and nurturing environment? (2) How useful are risk stratification tools in predicting poor outcomes? (3) Does a dedicated mental health ED improve the response to treatment for a young person with mental health concerns? (4) What is the effect of follow‐up services on the likelihood of returning to the ED with mental health concerns?

**TABLE 2:** Key data points identified.

Baseline data	% rated 7 or more	Outcomes—during ED visit	% rated 7 or more	Outcomes—postdischarge	% rated 7 or more
Family violence	96	Use of pharmacological restraint—injectable medication	92	Return to ED with the same problem	92
Child abuse	96	Behavioral disturbance/security response called	92	Suicide attempt	92
Childhood trauma	93	Severity of risk of self‐harm and/or suicide	88	Frequency of ED presentations over the next 12 months	80
Out‐of‐home/foster care	89	Use of physical restraint (being held by another person)	84	Engagement with community	80
Drug use	81	Time taken until reviewed by mental health professional	84	Death due to suicide	79
Child protection involvement	81	Patient feeling safe in ED	83	Attendance to follow‐up appointments	76
Alcohol use	81	Level of distress of patient	83	Providers the patient was referred to postdischarge	76
Other mental health conditions	80	Severity of suicidal thoughts	83	Waiting time until appropriate follow‐up^a^	68 (91)
Other neurodevelopmental condition (autism/ADHD/intellectual disability)	76	Frequency of suicidal thoughts	83		
		Use of mechanical restraint (shackles)	80		
		Use of chemical restraint—oral medication	80		
		Self‐harm occurring in ED	80		
		Time taken to be assessed	80		
		New referrals made in ED	80		
		Assessed by mental health professional with pediatric experience	79		
		Length of stay in ED	72		
		Staff availability	71		

*Note*: The consensus research outcomes and key data points.

Abbreviation: ADHD, attention deficit hyperactivity disorder.

^a^ Although this item has a consensus rating of 68 in Stage 3 surveys, it was classified as inconclusive and rescored during Stage 4 where 91 respondents were allocated a Likert score of 7 or higher.

The most important baseline data points included risk factors for mental health (Table 3). The top three listed baseline data points were family violence, child abuse, and childhood trauma.

**TABLE 3:** Research questions.

	Research question—top 20 highest rated research questions/themes	% rated 7 or more
1	What is required for an ED to be considered a safe and nurturing environment?^a^	100
2	How useful are risk stratification tools in predicting poor outcomes?^a^	100
3	Does a dedicated mental health ED improve the response to treatment for a young person with mental health concerns?	90
4	What is the effect of follow‐up services on the likelihood of returning to the ED with mental health concerns?	90
5	What is the relationship between prolonged waiting times and behavioral disturbance/distress?	86
6	Does a dedicated mental health ED improve the care provided to a young person with mental health concerns?	86
7	Does a dedicated mental health ED improve the patient experience of a young person with mental health concerns?	86
8	Does a purpose‐designed “low sensory” waiting room make it easier for young people to wait to see a mental health clinician?	86
9	Can patients be managed in their own homes instead of ED?	85
10	Should an Aboriginal liaison officer be involved in Aboriginal and/or Torres Strait Islander people's needs?	83
11	Which coping strategies are effective for young people to manage their own distress/aggression/anxiety?	82
12	What are the most effective ways of calming a young person with autism in the busy, loud setting of an emergency department?	82
13	Which nondrug strategies are most effective for the management of acute behavioral disturbance?	82
14	What aspects of the physical environment of the ED contribute (positively or negatively) to the care of a young person with mental health concerns?	81
15	What are the predictors of suicide in young people?	81
16	Is there a relationship between repeated ED visits and lack of availability of inpatient beds?	80
17	Which interventions and/or follow‐up services are most effective at reducing the likelihood of returning to the ED with mental health concerns?	80
18	Can primary and high schools assist with early detection, early intervention, and management of young people with underlying mental health issues?	80
19	What additional needs do young people with cultural and linguistic diversity have when they present to the ED with mental health concerns?	78
20	Why do people become agitated and/or aggressive while in the ED?	77

*Note*: The top 20 highest rated research questions based on proportion of respondents rating 7–9 on the Likert scale.

^a^ Questions were inconclusive in Stage 3 and hence included for prioritization in Stage 4.

The highest priority outcomes (Table 3) included the use of pharmacological restraint, behavioral disturbance during the ED visit, and likelihood of re‐presentation and/or suicide attempt following ED discharge. The top three listed outcomes occurring within an ED visit were (1) “Use of pharmacological restraint”; (2) “Was there behavioural disturbance/security response called?”; and (3) “How severe is the young person's risk of self‐harm and/or suicide?” The top three listed outcomes occurring after an ED visit were (1) “Did the young person return to the ED with the same problem?”; (2) “How many times did the young person return to the ED with the same problem?” and (3) “Suicide attempt.”

## DISCUSSION

Despite the rapid increase in child and adolescent mental health presentations in recent years, existing PEM network research prioritization exercises have rarely addressed this population.^9^, ^10^, ^12^, ^14^ Our study has, for the first time internationally, generated and prioritized a consensus list of future research questions/themes and key data points (baseline data and outcomes) related to pediatric emergency mental health presentations. Our findings will inform future prospective observational studies and randomized controlled trials in this population, and provide a clear direction for the development of specific, psychometrically sound patient‐reported outcome measures.^20^

Importantly, the prioritization process involved patients and parents/carers, those involved in prehospital care (police officers and ambulance paramedics), and emergency and mental health clinicians working in the ED. Notably, of the six global PEM research prioritization exercises in the past 8 years, to our knowledge, this is only the second study to involve families and the first PEM study to do so in Australia.^8^, ^9^, ^10^, ^11^, ^12^, ^13^, ^14^

One of the primary themes of the research questions concerned the suitability and safety of the ED environment for pediatric patients presenting with a mental health crisis. Despite encouragement to use specialized mental health services, deficits in mental health funding have left these services difficult to access for mental health patients.^21^ As a consequence, the ED has had to carry an increased burden as the primary point of access to health care for this vulnerable group, despite being somewhat ill‐equipped to do so.^21^, ^22^ The emphasis on “safety” and multiple references to “dedicated mental health EDs” in the consensus list of research questions highlights our participants' perception that the current ED model in Australia is not the ideal place to treat these patients and appears to reflect a desire for greater accessibility to specialized emergency pediatric mental health services and a greater focus on prevention to reduce the need for acute ED attendance.

Another primary research question highlighted by participants concerned the usefulness of risk stratification tools in predicting poor outcomes in patients with mental health concerns. A key component of any clinical encounter is to stratify risk; this information then guides further treatment decisions.^23^ However, there is considerable uncertainty regarding the optimal approach to clinical risk stratification. In recent years, there has been an interest in machine learning models to predict self‐harm^24^ and a predictive model applied to electronic medical record data outperformed a clinician‐administered 18‐point risk factor checklist for suicide at 180 days following discharge.^25^

Other common themes for the top research questions included management and prevention of behavioral disturbance within the ED, staff confidence and training, and the effects of follow‐up services on re‐presentation—again reflecting perceptions regarding the limitations of the current role EDs play for the pediatric mental health patient.

Despite the initial survey explicitly prompting research questions on specific mental health diagnoses (such as eating disorders, anxiety, and neurodevelopmental disorders) only one of the top 20 questions refers to a specific mental health presentation (calming a child with autism spectrum disorder in a busy ED).

In terms of outcome measures, many of the highly rated items related to the ED experience and treatment within the ED. This included restrictive interventions (e.g., pharmacological and physical restraints and security responses) during ED treatment. Perhaps surprisingly, protocol‐ and system‐based outcomes—such as *waiting time* and ED *length of stay*—were not rated highly, despite making up a large proportion of the content of the research questions (Table 2). Psychosocial determinants of mental health disorders such as childhood trauma, family violence, and a history of child abuse feature as the most highly rated items in the *Baseline Data* section (Table 2). Adverse childhood experiences (ACEs), such as childhood trauma, abuse, neglect, and community factors including poverty, are known to increase the risk of suicide and chronic mental health disorders,^26^ and the responses of our participants highlight the growing understanding of the contribution of cultural and societal factors to mental health presentations.^27^ However, detailed exploration of these issues may not be possible in a relatively brief routine ED encounter. It will be important to determine the optimal ways to ensure these issues are explored in a sensitive manner at an appropriate time in the ED visit, reliable data collection, and appropriate referral/response.^28^ To further help these patients coordinated support is required across health, education, and social care sectors.^29^

In previous PEM Delphi studies, research questions relating to mental health have been inconsistently represented in the finalized consensus. A similar Delphi process organized by the Pediatric Emergency Research Canada (PERC) network identified “mental health presentations” as its top priority for future research.^8^ Notably, the PERC study is the only prioritization study that included consumers. A prioritization study from the United States ranked “mental health presentations” as seventh on their list of priorities,^11^ while the Australia/New Zealand (PREDICT) Network and the Pediatric Emergency Research in the UK and Ireland (PERUKI) Delphi studies did not identify any mental health–related research questions as priorities when conducted in 2016 and in 2012, respectively.^9^, ^10^

## STRENGTHS AND LIMITATIONS

This is the first Australian study to systematically prioritize a consensus list of research topics and key data points in children and adolescents presenting to the ED with mental health concerns. It is the first pediatric ED study to involve Australian patients and carers to set research priorities.

In our study, one‐third of patient/carer participants had primary presentations of suicidality/self‐harm, which is broadly representative of patterns of ED mental health presentations for children and adolescents in Australia.^30^ Although we recruited from 12 hospitals over a 2‐month period, it is possible that this relatively brief window may have impacted the number and type of patients and care providers. In the future, purposeful sampling and enrollment of a wider variety of mental health and neurodevelopmental disorder presentations may be needed to generate research questions for specific groups of mental health patients, such as those presenting with eating disorders, psychotic illnesses, attention deficit hyperactivity disorder (ADHD) and autism spectrum disorder or substance abuse and their psychosocial and cultural context.

Despite emphasizing the importance of participation in all rounds during our recruitment phase and sending out two reminders for each stage, there was a high level of attrition in the number of respondents across the study, particularly with patients and parents/carers. This places our study at risk of bias and the possibility that our results are not fully representative of the intended study population. In addition to the impact of the COVID‐19 pandemic in general, a delay in survey collation across all sites led to a prolonged interval between the first and second survey rounds, which may have reduced the willingness of participants to complete following rounds of surveys. The second survey was also lengthy (with over 250 questions), which again may have influenced attrition. This should perhaps be addressed in future studies with greater incentives to complete each round of surveys and more timely intervals between surveys or a more efficient process such as the nominal group technique, which can be completed in a single day.^31^ It is possible that the disproportionate attrition of patients and parents/carers has biased our results toward views of clinicians.

Of the 36 patients/carers enrolled in the study, 50% identified as “Australian,” with three (8.3%) of that group identifying as “Australian Aboriginal.” As noted above, the Aboriginal and Torres Strait Islander populations in Australia are overrepresented in the distribution of pediatric mental health presentations,^21^ and it is unlikely that the results of this project adequately represents the views and experience of Aboriginal and Torres Strait Islander people. Similarly, due to the conduct of the surveys in English only, we should not consider our findings representative of culturally and linguistically diverse community members, including refugee populations.

Health care delivery and experiences depend on local resources and the organizational context of the broader system. The results of our study may not necessarily be generalizable across all EDs in Australia (e.g., rural and regional vs. urban settings) and unlikely to be directly applicable to other settings, health care systems, or countries around the world.

## CONCLUSIONS

Through the collaboration of both care providers and care recipients (patient/carers), a consensus of prioritized research questions and key data points regarding pediatric emergency mental health presentations has been identified. The results should be used to inform future pediatric research among Australian emergency centers and highlight important priority areas such as efficacy of dedicated mental health EDs and the effect of follow‐up services on re‐presentation rates. We aim to use this list of questions to focus research across Australia and Aotearoa New Zealand to improve ED mental health care and improve outcomes for this important population.

## AUTHOR CONTRIBUTIONS

Edmund Proper, Simon S. Craig, Marietta R. John‐White—drafting of manuscript. Simon S. Craig, Frank Muscara, Franz E. Babl, Vicki A. Anderson, Catherine L. Wilson, Meredith L. Borland, Bruce J. Tonge, Kylie M. Gray, Glenn A. Melvin, Rohan Borschmann, Richard Haslam, Emma J. Tavender, Michael S. Gordon, Stuart R. Dalziel, Amit Kochar, Marietta R. John‐White—concept and design, analysis and interpretation of data. Simon S. Craig, Karen Smith—interpretation of data. Simon S. Craig, Franz E. Babl, Meredith L. Borland, Amit Kochar, Marietta R. John‐White—acquisition of the data. Simon S. Craig, Frank Muscara, Franz E. Babl, Vicki A. Anderson, Catherine L. Wilson, Meredith L. Borland, Bruce J. Tonge, Kylie M. Gray, Glenn A. Melvin, Rohan Borschmann, Richard Haslam, Emma J. Tavender, Michael S. Gordon, Stuart R. Dalziel, Amit Kochar—critical revision of the manuscript for important intellectual content, statistical expertise. Simon S. Craig, Glenn A. Melvin, Franz E. Babl—acquisition of funding.

## FUNDING INFORMATION

This project was funded by a grant from the Australian Government Medical Research Future Fund (MRFF) Million Minds Mission (GNT1179137). The PREDICT network is funded by a Centres of Research Excellence grant from the National Health and Medical Research Council (GNT1171228). Viet Tran, Royal Hobart Hospital, Tasmania—the Royal Hobart Hospital Research Foundation Incorporated (Grant ID: 20‐205). Ben Lawton and Brook Charters, Logan Hospital, Queensland—2020 Metro South Health Research Support Scheme (MSH RSS; Grant ID RSS_2020_066). Rohan Borschmann receives salary and research support from a National Health and Medical Research Council Emerging Leadership‐2 Investigator Grant (NHMRC EL2; 2008073). Franz Babl received support from a National Health and Medical Research Council Investigator Grant, Canberra, Australia, and the Royal Children's Hospital Foundation, Parkville, Australia.

## CONFLICT OF INTEREST STATEMENT

The authors declare no conflicts of interest.

## Data Availability

The data that support the findings of this study are available from the corresponding author upon reasonable request.
